# Negative Predictive Value of a Prostate MRI in Black Men: Implications for Biopsy Decision-Making

**DOI:** 10.1097/JU.0000000000004498

**Published:** 2025-03-17

**Authors:** Sarah Sandlow, Samuel Carbunaru, Zequn Sun, Bernice Ofori, Yingzhe Liu, Eric Li, Courtney M. P. Hollowell, Patricia Vidal, Kent T. Perry, Hiten D. Patel, Ryan W. Dobbs, David D. Casalino, Cecil G. Wood, Anugayathri Jawahar, Channa Amarasekera, Jeanne M. Horowitz, Frank H. Miller, Emrah Gumusgoz, Ximing J. Yang, Bonnie Choy, Frances Manosca, Ulas Bagci, Edward M. Schaeffer, Peter Gann, Dustin D. French, Ashley E. Ross, Shilajit D. Kundu, Adam B. Murphy

**Affiliations:** 1 Department of Urology, Northwestern University Feinberg School of Medicine, Chicago, Illinois; 2 Department of Urology, NYU Langone School of Medicine, New York, New York; 3 Department of Preventive Medicine, Northwestern University Feinberg School of Medicine, Chicago, Illinois; 4 Division of Urology, Department of Surgery, Cook County Hospital, Chicago, Illinois; 5 Department of Radiology, MedStar Union Memorial Hospital, Hollywood, Maryland; 6 Department of Radiology, Northwestern University Feinberg School of Medicine, Chicago, Illinois; 7 Department of Pathology, Northwestern University Feinberg School of Medicine, Chicago, Illinois; 8 Department of Pathology, Cook County Hospital, Chicago, Illinois; 9 Department of Pathology, University of Illinois at Chicago, Chicago, Illinois; 10 Department of Ophthalmology and Medical Social Science, Northwestern University Feinberg School of Medicine, Chicago, Illinois

**Keywords:** prostate cancer, magnetic resonance imaging, African American men

## Abstract

**Purpose:**

Prostate Imaging Reporting and Data System (PIRADS) v2.1 scoring with multiparametric (mp) MRI has a pooled 90% negative predictive value (NPV). PSA density (PSAD) ≤ 0.15 ng/mL/cm^3^ has been shown to enhance mpMRI’s NPV in non-Black men. Populations with higher disease prevalence are known to have lower NPVs. Given that Black men have higher prostate cancer prevalence, we evaluate sensitivity and NPV of mpMRI in Black vs non-Black men to assess possible mpMRI performance differences. As an exploratory objective, we investigate PSAD thresholds providing ≥ 90% sensitivity in Black men.

**Materials and Methods:**

We prospectively recruited Black and non-Black men referred to outpatient urology clinics for abnormal PSA or prostate examination in 3 similar biomarker validation studies from 2017 to 2023 before MRI-informed diagnostic prostate biopsy. We combined the research cohorts with a retrospective clinical cohort of clinically similar Black and non-Black men from 1 academic institution who also underwent mpMRIs and MRI-informed biopsies. MRIs were scored using PIRADS version 2.0 or 2.1.

**Results:**

Our analysis included 286 Black men and 965 non-Black men with PSA ≤ 15.0 ng/mL. PIRADS < 3 had an NPV of 77.1% vs 87.6% and a sensitivity of 90.7% vs 96.3% for Gleason grade group 2 to 5 prostate cancer in Black vs non-Black men, respectively (both *P* < .05). Using PSAD ≥ 0.09 for Black men with PIRADS 1 to 2 lesions increased sensitivity to 92.9%.

**Conclusions:**

PIRADS < 3 has a lower NPV and sensitivity in Black men. For negative prostate MRIs, PSAD ≥ 0.09 may be a better threshold for safe biopsy deferral in Black men to maintain a ≥ 90% sensitivity.

Prostate cancer (PCa) is the second leading cause of cancer-related deaths among men worldwide.^1^ While PSA screening is widely used for PCa detection, its low specificity often leads to unnecessary prostate biopsies and overdiagnosis of Gleason grade group (GG) 1 PCa, prompting exploration of secondary screening modalities.^2,3^

**JU Insight**

**Study Need and Importance**
Prostate cancer (PCa) is a major cause of cancer-related deaths worldwide. While PSA screening is widely used, its low specificity leads to unnecessary biopsies and overdiagnosis of low-risk PCa. Multiparametric (mp) MRI using the Prostate Imaging Reporting and Data System (PIRADS) helps improve risk assessment and reduce unneeded biopsies. However, its effectiveness in high-risk Black men is unclear. Since Black men have a higher prevalence of clinically significant (cs) PCa, evaluating mpMRI’s accuracy in this group is essential for improving biopsy decision-making.
**What We Found**
Our study found that mpMRI at a PIRADS ≥ 3 threshold has lower sensitivity and negative predictive value for detecting csPCa in Black men compared with non-Black men. Black men had a higher risk of csPCa at PIRADS 1 to 4 scores, indicating poor calibration (see Figure). We also assessed PSA density (PSAD) thresholds to refine biopsy recommendations. Results suggest that the commonly used PSAD ≥ 0.15 threshold may be too high for safely deferring biopsies in Black men with PIRADS 1 to 3 scores. These findings highlight the need for race-specific clinical considerations.
**Limitations**
This study has limitations, including potential selection bias because of different research trial populations and a single academic medical center’s clinical cohort. Additionally, using radiology concordance conferences instead of centralized reviews may introduce variability in PIRADS scoring. Other factors influencing csPCa detection, such as family history, were not extensively analyzed.
**Interpretation for Patient Care**
Our findings highlight the importance of validating diagnostic tools in high-risk Black men. Given the lower negative predictive value of mpMRI, clinicians should consider using lower PSAD thresholds when making biopsy decisions. Including diverse populations in research is essential to ensuring precise prostate cancer screening and management.

**Figure.**
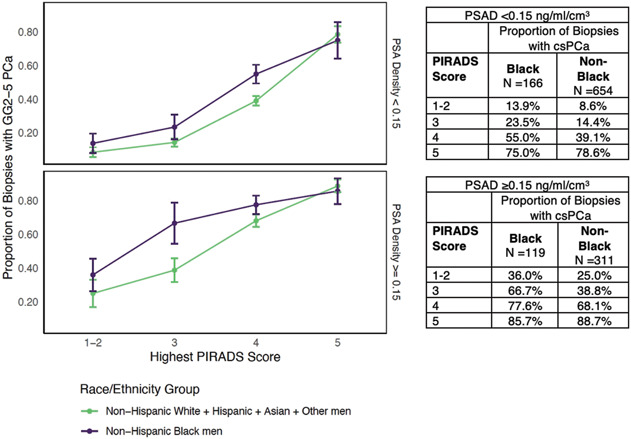
Proportion of clinically significant (cs) prostate cancer (PCa) vs Prostate Imaging Reporting and Data System (PIRADS) score in Black vs non-Black men. Proportion of Gleason grade group (GG) 2 to GG5 PCa vs PIRADS score is compared between non-Black and Black men and stratified by PSA density (PSAD) < 0.15 vs ≥ 0.15 ng/mL/cm^3^ shown with line graphs. Black men have higher rates of GG2 to GG5, or cs, PCa at PIRADS scores 1 to 4 in both PSAD strata, with proportions shown in the corresponding tables. Error bars represent SD of each proportion.

Multiparametric (mp) MRI of the prostate using the Prostate Imaging Reporting and Data System (PIRADS) is used for PCa detection and risk stratification.^4^ A negative MRI (PIRADS <3) indicates low likelihood of clinically significant (cs) PCa, defined as GG 2 to 5, and serves as a triage tool to defer biopsies.^5,6^ Pooled analyses estimate MRI’s negative predictive value (NPV) at > 90%^7^ with variation across populations. Because disease prevalence influences predictive values,^8^ NPVs are lower in populations with higher PCa risk, such as Black men, who tend to have more aggressive disease.^9^ Given mpMRI’s role in biopsy deferral and in risk calculators for biopsy decision-making,^10^ its efficacy in Black men warrants investigation.

This study compared mpMRI’s ability to predict csPCa in Black vs non-Black men by evaluating sensitivity and NPV at the PIRADS ≥ 3 threshold. We additionally assessed the impact of the commonly used PSA density (PSAD) < 0.15 ng/mL/cm^3^ threshold, identifying PSAD thresholds with 90% NPV for GG 2 to 5 PCa detection in Black men with PIRADS 1 to 2 and PIRADS 3 lesions in exploratory post hoc analyses.

## Materials and Methods

### Study Design and Participants

**Figure 1.**
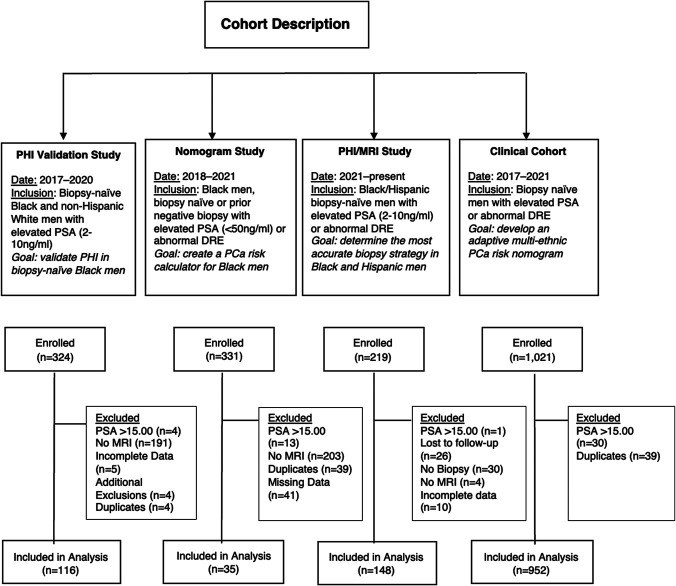
STARD (Standards for Reporting of Diagnostic Accuracy) chart. Three Institutional Review Board–approved research cohorts with similar inclusion criteria (Prostate Health Index [PHI] validation, nomogram, PHI/MRI) and 1 clinical registry cohort were combined for the final study population. DRE indicates digital rectal examination; PCa, prostate cancer.

We used data from an Institutional Review Board (IRB)–approved retrospective clinical cohort (IRB number STU00214996) and 3 IRB-approved prospective research cohorts (IRB numbers STU00204254, STU00205089, STU00212147), described in Figure 1. Informed consent was obtained for research cohorts. We included men with PSA ≤ 15.0 ng/mL who underwent prebiopsy mpMRI and MRI-informed prostate biopsy within 12 months (median 1, IQR 0-2 months) without prior PCa diagnoses.

The mpMRI studies were interpreted by general and genitourinary radiologists. Participating radiologists attended consensus conferences in December 2020 and April 2022, achieving > 90% concordance on PIRADS scoring. Approximately 50% of MRIs were read with PIRADS v2.0 while 50% were read with v2.1, which have nearly identical sensitivity^11^; most radiologists transitioned by December 2019. Our county hospital site used 1.5 T MRIs and university-affiliated sites used 3.0 T.

Urologists used transrectal or transperineal biopsy approaches, using UroNav MRI-US fusion transrectal ultrasound technology or cognitive freehand fusion with the Precision Point Transperineal Access System to obtain 2 to 3 cores per PIRADS 3 to 5 lesion, in addition to systematic 10 to 20 sextant or perineologic biopsy template cores. Participating pathologists assigned Gleason scores per the 2014 International Society of Urological Pathology guidelines^12^ and attended consensus conferences in December 2020 and April 2022, achieving > 80% concordance on GG scoring.

Demographic and clinical data were extracted from electronic medical records for the clinical cohort and from our study databases and electronic medical record for the research cohort. Self-reported race and ethnicity were used. Patients identifying as Hispanic or Latino, regardless of race, were included in the Hispanic/Latino ethnicity category. Owing to limited size, we combined non-Hispanic White men, Asian, Hispanic, and Other races into a “non-Black men” category, as sensitivity for GG 2 to 5 PCa detection at the PIRADS ≥ 3 threshold was similar across these groups (all ≥94%).

### Preliminary Data Analysis

Within the research cohort alone, we had 80% power to detect a 9.2% difference in NPV with a 2-sided ⍺ = .05. We combined cohorts to increase statistical power, after first using binary logistic regression and multiplicative interaction terms to ensure there was no effect modification of PIRADS’ association with GG 2 to 5 PCa diagnosis by study site, MRI magnet strength, cohort type, and Black vs non-Black race (all *P* > .10) Strong statistically significant associations were found for PIRADS as an ordinal variable (1-2, 3, 4, 5), PSAD ≥ 0.15 ng/mL/cm^3^, and Black race (all *P* < .001; data not shown).

Patient characteristics by cohort were summarized with descriptive statistics, using Pearson χ^2^ test and Fisher exact tests for categorical variables and Wilcoxon rank sum tests for continuous variables. Despite some statistical differences, both populations are clinically similar and represent contemporary biopsy cohorts. Calibration line plots of PIRADS score vs proportion of biopsies with GG 2 to 5 PCa between cohorts are shown in Supplemental Figure 1 (https://www.jurology.com). Both showed similar trends across PIRADS categories.

### Statistical Analysis

After combining cohorts, descriptive statistics were used to summarize demographic and clinical characteristics by race. Line plots were constructed to compare proportions of GG 2 to 5 PCa by PIRADS score for Black vs non-Black patients, stratified by PSAD ≥ 0.15 ng/mL/cm^3^ threshold for calibration. We computed sensitivity, specificity, positive predictive value, and NPV of the PIRADS ≥ 3 threshold to compare the overall accuracy.

We had 80% power to detect a 5.0% difference in NPV and a 5.6% difference in sensitivity between Black and non-Black men. We assumed 90% NPV and 87% pooled sensitivity for GG 2 to 5 PCa detection using mpMRI in non-Black men with a 2-sided ⍺ = .05.^13^

Our exploratory post hoc analysis used race-stratified 2 × 2 contingency tables to assess PSAD thresholds in 0.01 increments between 0.05 and 0.15, to maximize specificity while achieving sensitivities ≥ 90% for detecting csPCa in Black men with negative and equivocal MRIs.

## Results

Our analysis included 1251 patients: 299 research cohort patients and 952 clinical cohort patients. Supplemental Table 1 (https://www.jurology.com) shows a larger percentage of Black patients (65.0% vs 9.8%, *P* < .001) in the research cohort and differences in PIRADS score distributions (*P* < .001). As expected, more research cohort patients had negative MRIs compared with clinical cohort patients (27.0% vs 11.0%, *P* < .001) because of protocolized MRIs and biopsies in the former. The research cohort had a lower proportion of GG 2 to 5 PCa diagnoses compared with the clinical cohort (38.0% vs 46.0%, *P* = .02).

Table 1 compares population characteristics between Black and non-Black participants. Black patients had higher PSAD, more abnormal digital rectal examinations, and more negative MRIs (all *P* ≤ .001). Other statistically significant differences included age and serum PSA levels, although the measures were similar and are representative of modern biopsy cohorts. Differences were found in GG 2 to 5 PCa detection rates: 53.0% in Black men vs 42.0% in non-Black men (*P* = .001).

**Table 1.** Clinical and Demographic Characteristics by Race Group

Characteristics	Black (n = 286)	Non-Black (n = 965)	*P* value
Age, median (IQR), y	62 (56, 68)	63 (57, 69)	.03
PSA, median (IQR), ng/mL	5.93 (4.49, 7.98)	5.16 (4.06, 7.02)	< .001
PSA density, median (IQR), ng/mL/cm^3^	0.13 (0.09, 0.21)	0.11 (0.08, 0.17)	< .001
Prostate volume, median (IQR), cm^3^	42 (30, 65)	46 (34, 64)	.051
Family history of PCa, No. (%)	60 (21.0)	210 (22.0)	.78
Abnormal DRE, No. (%)	32 (12.0)	56 (5.8)	< .001
Prior prostate biopsy, No. (%)	25 (8.7)	32 (3.4)	< .001
PIRADS score, No. (%)			
1-2	62 (22.0)	122 (13.0)	< .001
3	49 (17.0)	250 (26.0)	
4	138 (48.0)	452 (47.0)	
5	37 (13.0)	141 (15.0)	
Biopsy technique, No. (%)			
Transrectal	62 (22.0)	109 (11.0)	< .001
Transrectal (fusion)	146 (51.0)	777 (81.0)	
Transperineal	8 (2.8)	14 (1.5)	
Transperineal (fusion)	70 (24.0)	65 (6.7)	
Biopsy results, No. (%)			
Negative	87 (30.0)	424 (44.0)	.003
GG1	48 (17.0)	136 (14.0)	
GG2-5	151 (53.0)	405 (42.0)	
Race/ethnicity, No. (%)			
White	0 (0.0)	731 (75.8)	
Black	286 (100)	0 (0.0)	
Hispanic	0 (0.0)	79 (8.2)	
Asian	0 (0.0)	21 (2.2)	
Other	0 (0.0)	134 (13.9)	

Abbreviations: DRE, digital rectal examination; GG, Gleason grade group; PCa, prostate cancer; PIRADS, Prostate Imaging Reporting and Data System.

Wilcoxon rank sum tests were used to compare continuous variables, and χ^2^ tests and Fisher exact tests were used for categorical variables.

Black vs non-Black participants’ characteristics within PIRADS 1 to 3 mirrored the distribution of statistically significant differences in the overall cohort (analysis not shown); Black men also had higher PSA, PSAD, and frequency of prior negative biopsy and negative MRI (all *P* < .03).

**Figure 2.**
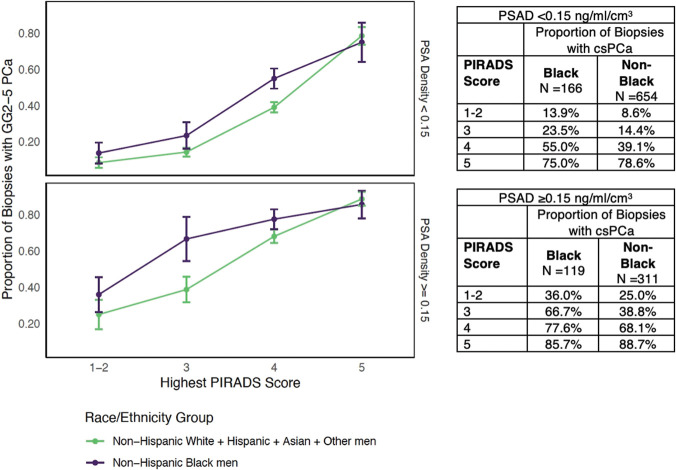
Proportion of clinically significant (cs) prostate cancer (PCa) vs Prostate Imaging Reporting and Data System (PIRADS) score in Black vs non-Black men. Proportion of Gleason grade group (GG) 2 to GG5 PCa vs PIRADS score is compared between non-Black and Black men and stratified by PSA density (PSAD) < 0.15 vs ≥ 0.15 ng/mL/cm^3^ shown with line graphs. Black men have higher rates of GG2 to GG5, or cs, PCa at PIRADS scores 1 to 4 in both PSAD strata, with proportions shown in the corresponding tables. Error bars represent SD of each proportion.

Figure 2 shows the proportion of GG 2 to 5 PCa detection by PIRADS score, stratified by PSAD ≥ 0.15 ng/mL/cm^3^. Black men have higher proportions of GG 2 to 5 PCa than non-Black men across PIRADS 1 to 2, 3, and 4 scores, with similar proportions for PIRADS 5. Among Black men with PSAD ≥ 0.15 and PIRADS 1 to 2 MRIs, 36.0% had GG 2 to 5 PCa vs 25.0% in non-Black men. For PSAD < 0.15, 13.9% of Black men with PIRADS 1 to 2 had GG 2 to 5 PCa vs 8.6% of non-Black men. Similar trends are observed for PIRADS 3 lesions both at PSAD ≥ 0.15 (66.7% Black men with csPCa vs 38.8% non-Black men) and PSAD < 0.15 (23.5% Black men vs 14.4% non-Black men).

Table 2 provides sensitivity, specificity, NPV, and positive predictive value of the PIRADS ≥ 3 threshold by race, along with this threshold’s biopsy outcome distribution. Significant differences were observed between Black and non-Black men for sensitivity (90.7% vs 96.3%, *P* = .004) and NPV (77.1% vs 87.6%, *P* = .03). Among men with negative MRIs, Black men had an absolute difference of 26.2% fewer negative biopsies relative to non-Black men and a relative 83.7% increase in csPCa (*P* = .019).

**Table 2.** Comparison of Sensitivity and Negative Predictive Value and Biopsy Outcomes of the Prostate Imaging Reporting and Data System ≥ 3 Threshold by Race

Race/ethnic group	Sensitivity (%)	Specificity (%)	PPV (%)	NPV (%)	No. patients biopsied (%)	No. cancers missed (%)	No. cancers detected (%)
Clinical cohort: PIRADS ≥3 threshold							
Non-Black (N = 859)	96.1	16.8	48.0	84.2	764 (88.9)	15 (3.9)	367 (96.1)
Black (N = 93)	93.2	11.8	64.7	50.0	85 (91.4)	4 (6.8)	55 (93.2)
Research cohort: PIRADS ≥3 threshold							
Non-Black (N = 106)	100.0	31.7	29.1	100	79 (75.2)	0 (0.0)	23 (100)
Black (N = 193)	89.1	43.0	59.0	81.1	139 (72.4)	10 (10.9)	82 (89.1)
Combined cohort: PIRADS ≥ 3 threshold							
Non-Black (N = 955)	96.3^a^	19.0	46.3	87.6^a^	843 (87.4)	15 (3.7)	390 (96.3)
Black (N = 286)	90.7	35.1	61.2	77.1	224 (78.6)	14 (9.3)	137 (90.7)

PIRADS 1-2 biopsy outcomes	Non-Black N = 122, %	Black N = 62, %	*P* value
Negative biopsy	71.3	45.2	.02^b^
GG1	16.4	32.3	
GG2	9.0	17.7	
GG3	3.3	3.2	
GG4	0.0	1.6	
GG5	0.0	0.0	

Abbreviations: GG, Gleason grade group; NPV, negative predictive value; PIRADS, Prostate Imaging Reporting and Data System; PPV, positive predictive value.

a For the main outcomes, sensitivity (*P* = .004) and NPV (*P* = .03) were statistically different between non-Black and Black men in the combined cohort. χ^2^ tests were used to compare sensitivity and NPV (proportions) by race.

b A χ^2^ trend test was used to assess for statistical significance for biopsy outcomes (*P* = .02).

Supplemental Table 2 (https://www.jurology.com) displays the post hoc analysis assessing sensitivity and NPV of different PSAD thresholds to identify one with > 90% sensitivity for negative and equivocal mpMRIs in Black men. In our study, the commonly used PSAD < 0.15 threshold had 64.3% sensitivity and 86.1% NPV in Black men with negative MRIs. A PSAD < 0.09 increased sensitivity to 92.9% and NPV to 94.4%. The specificity, a proxy for avoidable biopsies, of PSAD < 0.09 was 36.2%.

In Black men with PIRADS 3 lesions, PSAD < 0.15 had a 55.6% sensitivity and a 76.5% NPV (*P* = .004). A PSAD < 0.07 threshold achieved 88.9% sensitivity, 80.0% NPV, and 25.8% specificity.

We calculated sensitivity and NPV for 2 biopsy deferral strategies using PIRADS and PSAD thresholds in series. In Black participants, using PIRADS < 3 followed by PSAD < 0.15 had a 91.4% sensitivity, an 81.4% NPV, and a 42.5% specificity. Using PSAD < 0.09 for PIRADS 1 to 2 and PSAD < 0.07 for PIRADS 3 yielded 98.0% sensitivity, 89.3% NPV, and 18.7% specificity.

## Discussion

Our comparative effectiveness study assessed mpMRI’s performance, finding a significantly lower NPV in Black men (77.1%) compared with non-Black men (87.6%). Our exploratory analysis stratifying negative MRIs by PSAD found a PSAD < 0.15 threshold still missed > 10% of GG 2 to 5 PCa in Black men, with an 86.1% NPV in PIRADS 1 to 2. Within PIRADS 1 to 2 scores, Black men had a 91.1% higher risk of cancer on biopsy relative to non-Black men. While much of this is indolent (16% GG 1 increase), there is still a 10% higher csPCa rate in Black men (see Table 2). This suggests that the published literature’s reported probabilities of csPCa in PIRADS 1 to 2 scores may underestimate Black patients’ risk. Our post hoc analysis suggests that lowering the PSAD threshold from 0.15 to 0.09 for negative MRIs could reduce missed csPCa, allowing some men to safely avoid unnecessary biopsies.

Screening guidelines for PCa have widely adopted mpMRI for use.^14,15^ Many urologists now use mpMRI to identify candidates for biopsy, often deferring biopsy in patients with negative MRIs (PIRADS 1-2). The meta-analysis by Sathianathen et al of 40+ studies cited mpMRI’s NPV as ∼90%, from a population of over 7300 biopsy-naïve men from academic and community hospitals.^7^ The population’s racial breakdown was not reported. Our reported NPV of 87.6% in non-Black men is similar to other studies’ retrospective cohorts, including Ma et al’s^16^ NPV of 87% and Lo et al’s^17^ NPV of 86%. MRI’s NPV in Black men, a population historically at higher risk of harboring more aggressive disease,^18^ has not been well-studied. While some studies found no racial differences in mpMRI’s performance (n = 60 Black men),^19^ others reported lower NPVs in Black men with GG2 PCa (n = 139 Black men).^20^ Notably, Chatterjee et al’s^21^ study attributes mpMRI’s underperformance to a lower area under the curve for distinguishing cancer from benign tissue in apparent diffusion coefficient and T2-weighted MRI sequences in Black men.

Our study found MRI’s NPV in Black men to be 77.1%, meaning a negative MRI is inaccurate ∼23% of the time. If externally validated, negative MRIs should not be used alone in clinical practice to defer biopsy in Black men, suggesting a need for secondary screening tools. As seen in Table 1, despite higher biopsy rates after a negative prostate MRI (based on protocol and clinical suspicion), Black men still had higher csPCa rates than non-Black men (53% vs 42%, *P* = .003). Additional sensitivity analyses found similar results when limiting the PSA to ≤ 10 ng/mL and 4 to 10 ng/mL and when excluding 1.5 T MRI (data not shown). Interaction terms between clinical cohort and race and clinical cohort and PIRADS score were tested in binary logistic regression models for prediction of csPCa; none were statistically significant (*P* > .20).

We explored various PSAD thresholds in negative and equivocal MRIs that maintained ≥ 90% sensitivity as reported in the literature.^22^ Falagario et al^23^ found that using PIRADS < 3 and PSAD < 0.15 thresholds in series had 95% NPV. While several manuscripts suggest deferring biopsy for men with a PSAD < 0.15 for PIRADS 1 to 3,^24,25^ in our study, this threshold failed to maintain adequate NPV in Black men with PIRADS 1 to 3 lesions. We found that using PSAD < 0.09 for PIRADS 1 to 2 has an NPV and sensitivity > 90% to detect GG 2 to 5 PCa in Black men, while PSAD < 0.07 improves sensitivity and NPV for detecting GG 2 to 5 PCa in Black men with PIRADS 3 lesions, despite reduced specificity (25% to 36%). Combining prostate MRI and PSAD reduces overall specificity to 18.7%. Fewer Black men may be able to avoid prostate biopsies despite negative or equivocal prostate MRIs.

Prior research cites 70% to 78% specificity for the PSAD < 0.15 threshold.^24,26^ Our proposed strategy involves biopsying all PIRADS 4 to 5 lesions, deferring biopsy in PIRADS 1 to 2 if PSAD < 0.09, and deferring biopsy in PIRADS 3 if PSAD < 0.07. While this strategy has low specificity (18.7%), its high 98.0% sensitivity and 89.3% NPV suggest that Black men with negative or equivocal MRIs may need another biomarker to safely defer biopsy. Comparatively, using PSAD < 0.15 in our proposed strategy had lower sensitivity and NPV, at 91.4% and 81.4%, respectively. This suggests urologists should treat PIRADS 1 to 2 and PIRADS 3 lesions differently in biopsy decision-making. Given doubled rates of uninsured status among Black men relative to non-Hispanic White men, less care access, and higher loss to follow-up rates, maintaining high NPV may be more important for this population.^27,28^

Some limitations should be noted. To increase statistical power, we combined populations from studies with varying inclusion criteria. For generalizability, we recruited from community hospitals with 1.5 T MRIs (4.0%) and academic medical centers with 3.0 T MRIs (96.0%), using a PSA threshold of ≤ 15.0 ng/mL instead of 2 to 10 ng/mL. Notably, the PROMIS study used 1.5 T scanners and PSA ≤ 15.0 ng/mL, and the PRECISION trial also used 1.5 T and 3 T scanners with PSA ≤ 20 ng/mL. We modeled our inclusion criteria off both trials, which validated prostate mpMRI and MRI-targeted biopsy.^29,30^ We ran binary logistic regression models with csPCa as the outcome, finding that PIRADS scores, Black race, PSA density ≥ 0.15, and log_2_(PSA) were independent predictors in men with PSA ≤ 15.0. Sensitivity analyses assessing for effect modification of PIRADS by site, magnet strength, PSA levels, and cohort type showed no statistical significance.

We did not perform formal centralized review or assess MRI quality but minimized PIRADS scoring variances through consensus conferences, achieving > 90% concordance in PIRADS scoring. We also harmonized imaging protocols and reporting during the conferences. Higher PIRADS scores and the PSAD ≥ 0.15 ng/mL/cm^3^ threshold were strongly associated with increased GG 2 to 5 PCa likelihood, highlighting the value of combining imaging and serum biomarkers in the PCa diagnostic pathway. These findings, and higher odds of csPCa detection in Black men, emphasize the need for race-tailored screening strategies and ongoing investigation into racial disparities in detection.

Future research should externally validate these findings in multicenter prospective cohorts, given the potential implications on biopsy decision-making for Black men. Prospective studies powered to identify PSAD thresholds with adequate sensitivity and NPV must be conducted in Black men with PIRADS 1 to 3 lesions. There is a need to elucidate reasons for increased MRI-invisible csPCa in Black men and explore mpMRI accuracy in ethnic groups with lower PCa prevalence like Asians. While preliminary data suggest that apparent diffusion coefficient-weighted and T2-weighted imaging is less accurate in discriminating between PCa and benign tissue in Black men,^21^ this should be validated in larger populations.

## Conclusions

Our study suggests that prostate MRI has lower NPV and sensitivity in Black men with PSA ≤ 15.0 ng/mL. We posit that lower PSAD thresholds can be used with PIRADS 1 to 3 MRIs in Black men to maintain appropriate NPV.

## Supplementary Material
