# Supplementary material for: Negative Predictive Value of a Prostate MRI in Black Men: Implications for Biopsy Decision-Making
Source: J Urol. 2025 Mar 17;213(6):713–21. doi: 10.1097/JU.0000000000004498 (PMC12064359; doi:10.1097/JU.0000000000004498)
Supplement: Supplementary file 1 [file juro-213-713-s001.pdf]

Supplemental Figure 1: Proportion of Clinically Significant Prostate Cancer vs. PIRADS Score in the Research vs. Clinical Cohort Stratified by PSA Density

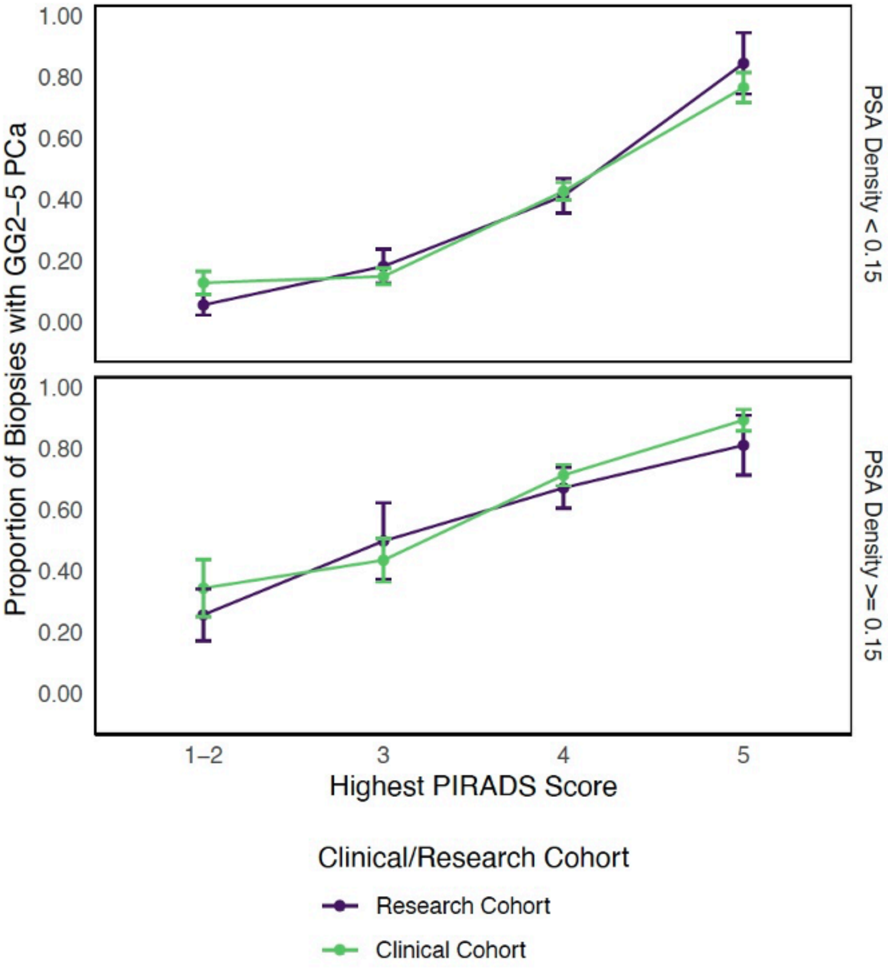

PSAD <0.15 ng/ml/cm³		
	Proportion of Biopsies with csPCa	
**PIRADS Score**	**Research Cohort** N =190	**Clinical Cohort** N =630
1-2	5.8%	13.0%
3	18.4%	15.1%
4	41.3%	42.9%
5	84.6%	76.7%

PSAD ≥0.15 ng/ml/cm³		
	Proportion of Biopsies with csPCa	
**PIRADS Score**	**Research Cohort** N =108	**Clinical Cohort** N =322
1-2	25.9%	34.6%
3	50.0%	43.8%
4	67.3%	71.5%
5	81.2%	89.5%
