# Supplementary material for: Negative Predictive Value of a Prostate MRI in Black Men: Implications for Biopsy Decision-Making
Source: J Urol. 2025 Mar 17;213(6):713–21. doi: 10.1097/JU.0000000000004498 (PMC12064359; doi:10.1097/JU.0000000000004498)
Supplement: Supplementary file 2 [file juro-213-713-s002.pdf]

Supplemental Table 1. Clinical and Demographic Characteristics by Cohort Group

**Characteristics**	**Research (n = 299)**	**Clinical (n = 952)**	**p-value**
Continuous variables	Median [IQR]	Median [IQR]	
Age, years	62 [56, 68]	63 [57, 69]	0.02
PSA, ng/ml	5.68 [4.22, 7.71]	5.25 [4.11, 7.18]	0.13
PSA Density, ng/ml/cm³	0.12 [0.08, 0.18]	0.12 [0.08, 0.17]	0.56
Prostate Volume, cm³	44 [32, 67]	46 [33, 63]	0.36
**Characteristics**	**Research (n = 299)**	**Clinical (n = 952)**	**p-value**
Categorical variables	N (%)	N (%)	
Family History of PCa, yes	71 (24.0%)	199 (21.0%)	0.25
Abnormal DRE, yes	42 (15.0%)	46 (4.8%)	p <0.001
**Prior Negative Prostate Biopsies**			
0	242 (81.0%)	952 (100.0%)	p <0.001
1	34 (11.0%)	0 (0.0%)	
>2	23 (7.7%)	0 (0.0%)	
**PIRADS Score**			
1-2	81 (27.0%)	103 (11.0%)	p <0.001
3	65 (22.0%)	234 (25.0%)	
4	124 (41.0%)	466 (49.0%)	
5	29 (9.7%)	149 (16.0%)	
**Biopsy Technique**			
Transrectal	80 (26.8%)	91 (9.5%)	p <0.001
Transrectal (fusion)	131 (43.8%)	792 (83.2%)	
Transperineal	10 (3.3%)	12 (1.3%)	
Transperineal (fusion)	78 (26.1%)	57 (6.0%)	
**Biopsy Results**			
Negative	125 (42.0%)	386 (41.0%)	0.004
GG1	59 (20.0%)	125 (13.0%)	
GG2-5	115 (38.0%)	441 (46.0%)	
**Race/Ethnicity**			
White (n =759)	75 (25.0%)	656 (69.0%)	
Black (n =286)	193 (65.0%)	93 (9.8%)	
Hispanic (n =79)	31 (10.0%)	48 (5.0%)	
Asian (n =21)	0 (0.0%)	21 (2.2%)	
Other (n =134)	0 (0.0%)	134 (14.0%)	
