# Supplementary material for: Negative Predictive Value of a Prostate MRI in Black Men: Implications for Biopsy Decision-Making
Source: J Urol. 2025 Mar 17;213(6):713–21. doi: 10.1097/JU.0000000000004498 (PMC12064359; doi:10.1097/JU.0000000000004498)
Supplement: Supplementary file 3 [file juro-213-713-s003.pdf]

Supplemental Table 2: Comparison of Sensitivity, Specificity, Positive Predictive Value and Negative Predictive Value by PSA Density Thresholds Across Race, by PIRADS Score

PIRADS 1-2				
	Black			
**PSAD cut off**	**Sensitivity**	**Specificity**	**PPV**	**NPV**
0.05	100.00	14.89	25.93	100.00
0.06	92.86	19.15	25.49	90.00
0.07	92.86	27.66	27.66	92.86
0.08	92.86	29.79	28.26	93.33
**0.09**	**92.86**	**36.17**	**30.23**	**94.44**
0.10	85.71	44.68	31.58	91.30
0.11	85.71	48.94	33.33	92.00
0.12	85.71	53.19	35.29	92.59
0.13	78.57	61.70	37.93	90.62
0.14	78.57	63.83	39.29	90.91
0.15	64.29	65.96	36.00	86.11

PIRADS 3				
	Black			
**PSAD cut off**	**Sensitivity**	**Specificity**	**PPV**	**NPV**
0.05	100.00	9.68	39.13	100.00
0.06	100.00	16.13	40.91	100.00
**0.07**	**88.89**	**25.81**	**41.03**	**80.00**
0.08	83.33	25.81	39.47	72.73
0.09	77.78	35.48	41.48	73.33
0.10	61.11	41.94	37.93	65.00
0.11	61.11	58.06	45.83	72.00
0.12	61.11	67.74	52.83	75.00
0.13	55.56	70.97	52.63	73.33
0.14	55.56	83.87	66.67	76.47
0.15	55.56	83.87	66.67	76.47
